# Autophagy in BRAF-mutant cutaneous melanoma: recent advances and therapeutic perspective

**DOI:** 10.1038/s41420-023-01496-w

**Published:** 2023-06-29

**Authors:** Elisabetta Fratta, Giorgio Giurato, Roberto Guerrieri, Francesca Colizzi, Jessica Dal Col, Alessandro Weisz, Agostino Steffan, Barbara Montico

**Affiliations:** 1 Immunopathology and Cancer Biomarkers Unit, Centro di Riferimento Oncologico di Aviano (CRO), IRCCS, Aviano, Italy; 2 Laboratory of Molecular Medicine and Genomics, Department of Medicine, Surgery and Dentistry “Scuola Medica Salernitana”, University of Salerno, 84081 Baronissi, SA Italy; 3 Genome Research Center for Health - CRGS, 84081 Baronissi, SA Italy; 4 Department of Medicine, Surgery and Dentistry “Scuola Medica Salernitana”, University of Salerno, 84081 Baronissi, SA Italy; 5 Molecular Pathology and Medical Genomics Program, AOU ‘S. Giovanni di Dio e Ruggi d’Aragona’ University of Salerno and Rete Oncologica Campana, 84131 Salerno, Italy

**Keywords:** Melanoma, Autophagy

## Abstract

Macroautophagy, hereafter referred to as autophagy, represents a highly conserved catabolic process that maintains cellular homeostasis. At present, the role of autophagy in cutaneous melanoma (CM) is still controversial, since it appears to be tumor-suppressive at early stages of malignant transformation and cancer-promoting during disease progression. Interestingly, autophagy has been found to be often increased in CM harboring BRAF mutation and to impair the response to targeted therapy. In addition to autophagy, numerous studies have recently conducted in cancer to elucidate the molecular mechanisms of mitophagy, a selective form of mitochondria autophagy, and secretory autophagy, a process that facilitates unconventional cellular secretion. Although several aspects of mitophagy and secretory autophagy have been investigated in depth, their involvement in BRAF-mutant CM biology has only recently emerged. In this review, we aim to overview autophagy dysregulation in BRAF-mutant CM, along with the therapeutic advantages that may arise from combining autophagy inhibitors with targeted therapy. In addition, the recent advances on mitophagy and secretory autophagy involvement in BRAF-mutant CM will be also discussed. Finally, since a number of autophagy-related non-coding RNAs (ncRNAs) have been identified so far, we will briefly discussed recent advances linking ncRNAs to autophagy regulation in BRAF-mutant CM.

## Facts

1. The role of autophagy in CM progression is still controversial.
2. Non-coding RNAs are emerging regulators of autophagy.
3. Autophagy inhibitors in combination with current drugs have been tested in clinical trials.

## Open questions

1. How does autophagy inhibition/activation affect tumor progression?
2. How does autophagy impact on mitochondria homeostasis in CM?
3. How does autophagy modulate CM microenvironment?
4. How do ncRNAs modulate autophagy?
5. What are the consequences of autophagy inhibition in light of the new treatment approaches in CM?

## Introduction

According to the latest classification of melanocytic tumors by the World Health Organization (WHO), melanomas can be subdivided into nine categories, with different characteristics and genetic background [1–4]. Notably, more than 90% of melanoma cases are cutaneous melanomas (CM), which usually arise from sun-exposed skin [5].

CM develops from the uncontrolled transformation of pigment-producing melanocytes and it is considered the most aggressive form of skin cancer worldwide with an increasing global incidence [6]. Early stage or primary CM are usually surgically resected, whereas advanced or metastatic CM still represents a therapeutic challenge. More recently, the increasing understanding of CM biology has resulted in the development of other therapeutic strategies, including the use of selective inhibitors of the mitogen-activated protein kinase (MAPK) signaling pathway, which is frequently dysregulated in CM patients due to activating mutations in the proto-oncogenes BRAF or NRAS, with a frequency of 50–70% and 15–30%, respectively. Among BRAF mutations, the V600E is the most common (90%), followed by the V600K (10–30%) [3, 7]. The constitutive activation of MAPK signaling, caused by mutant BRAF, appears a major driver of CM proliferation, survival and progression [8]. Interestingly, BRAF-mutation has also been described in other non CM, with the exception of uveal melanoma [9].

So far, small molecule inhibitors of BRAF (BRAFi), alone or in combinations with MEK inhibitors (MEKi), have demonstrated important clinical activities in BRAF-mutant CM, with remarkable response rates, and a significantly improved progression-free and overall survival in the advanced disease [10]. However, the clinical effectiveness of these targeted therapeutics is often greatly impaired by intrinsic or acquired drug resistance, which finally leads to CM progression [11–13].

## Autophagy in BRAF-mutant CM

Autophagy is an evolutionarily conserved process through which cellular contents, such as damaged organelles and protein aggregates, are delivered to lysosomes for degradation [14]. Several forms of autophagy have been described, including microautophagy, chaperone-mediated autophagy (CMA), and macroautophagy.

In microautophagy, which represents the less characterized form of autophagy, the lysosome or the endosome directly engulfs the cargo by protrusion or invagination. This process is mainly mediated by the endosomal sorting complexes required for transport and, partially, by the autophagy-related proteins (ATGs) [15]. CMA, the second type of autophagy, is a multi-step process that degrades target proteins containing a KFERQ-like motif that is recognized by the chaperone protein Hsc70. Protein internalization into the lysosome is then mediated by the binding between the target protein-Hsc70 complex and the lysosome-associated membrane protein type 2 A [16].

Of the three types of autophagy, macroautophagy is the most studied. Macroautophagy, hereafter referred to as autophagy, can be activated in response to several stimuli, such as nutrient deprivation, oxidative stress, hypoxia, or infection, and plays a cytoprotective or an adaptive role [17, 18]. Autophagy starts with the nucleation of a phagopore that engulfs material for degradation, thus expanding into a double-membraned organelle called autophagosome. Ultimately, the autophagosome fuses with the lysosome, and the cargo is finally degraded [18, 19].

**Fig. 1:**
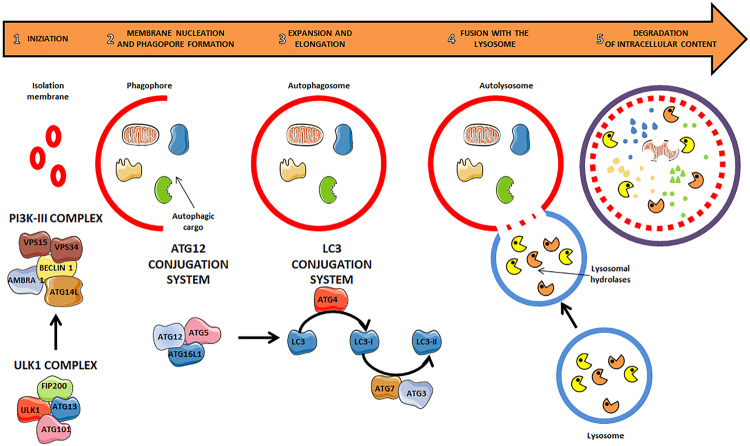
A schematic overview of autophagy pathway is shown. 1 Autophagy process starts with the formation of the ULK complex composed by ATG101, ATG13, ULK1 and FIP200 proteins. Once activated it stimulates the formation of the PI3K complex which is formed by AMBRA1, BECLIN1, ATG14L, VPS15 AND VPS34. This is associated with the membrane nucleation that is necessary for the formation of the autophagosome. 2 At the molecular level phagopore formation is associated with the formation of the ATG12 conjugation system. This system is formed by ATG12, ATG5 and ATG16L1that promotes LC3 conjugation. 3 The formation of the LC3 conjugation system allowes LC3 to be proteolitically cleaved by ATG4 in LC3-I, and then conjugated with phosphatidylethanolamine (PE) to form LC3-II. Meanwhile the phagopore elongates and LC3-II becomes necessary for cargo incorporation into the newly formed autophagosome. 4–5 Finally, the autophagosome fuses with the lysosome and the cargo is degraded by lysosomal enzymes. The figure was partly generated using Servier Medical Art, provided by Servier, licensed under a Creative Commons Attribution 3.0 unported license.

At the molecular level, autophagy is a multi-step process that consists of several phases: 1 initiation; 2 membrane nucleation and phagophore formation; 3 phagophore expansion and elongation; 4 fusion of the autophagosome with the lysosome; 5 degradation of intracellular content [20] (Fig. 1). The initiation of autophagy is characterized by the formation of the Unc-51 like autophagy activating kinase (ULK) complex, which consists of the ULK1 protein kinase itself, the FAK family kinase interacting protein of 200 kDa (FIP200), and the ATGs 13 and 101 [20, 21], which belong to a family of genes and proteins that are implicated in all stages of autophagosome formation [22]. Under normal conditions, ULK complex is inhibited by mechanistic target of rapamycin (mTOR), which phosphorylates ULK1 and ATG13. Once autophagy is activated, the mTOR-mediated inhibition of ULK is prevented, allowing the ULK complex to promote the formation of the class III-phosphoinositide 3-kinase (PI3K) complex. This complex is composed by several proteins, including beclin-1, which plays a central role in the autophagosome formation (for review see [23]). Beclin-1 is phosphorylated by ULK1 and recruits many proteins to induce the formation of the autophagosome. The subsequent phagophore expansion and elongation involve the microtubule-associated protein 1 light chain 3 (LC3) and a number of ATG proteins. Once synthesized, cytoplasmic LC3 is cleaved by a cysteine protease, ATG4B, to produce LC3-I, which exposes a C-terminal glycine that is then conjugated to phosphatidylethanolamine. This process occurs through a sequence of ubiquitination-like reactions that requires ATG7, ATG3, and the ATG12-ATG5-ATG16L1 complex. The lipidated form of LC3, LC3-II, associates with the autophagosome membrane, where it interacts with the adaptor protein sequestosome 1 (SQTM1, known as p62), which recognizes the ubiquitinated substrates, and targets them to the autophagosomes. In the final step, the autophagosomes fuse with lysosomes to form the autophagolysosomes, in which the cargo is degraded by lysosomal hydrolases [14, 20, 21, 24–26].

In the last years, several studies have documented high rates of autophagy in BRAF-mutant CM [27–29], and autophagy-related genes were shown to promote BRAF^V600^-mutant CM growth and survival [27, 30, 31]. More importantly, autophagy has been described as a major adaptive resistance mechanism to targeted therapies in BRAF^V600^-mutant CM, thus suggesting that autophagy inhibition might improve the response to BRAFi/MEKi [28]. Despite these findings, however, it is increasingly clear that autophagy defects might modulate BRAF-mutant CM growth in a bimodal manner. Indeed, autophagy promotes, but also suppresses, development and progression of BRAF-mutant CM, thus complicating therapeutic intervention. Based on these considerations, hereafter we will review the current knowledge on the ambiguous role of autophagy in BRAF-mutant CM.

### Pro-tumor role of autophagy in BRAF-mutant CM

**Fig. 2:**
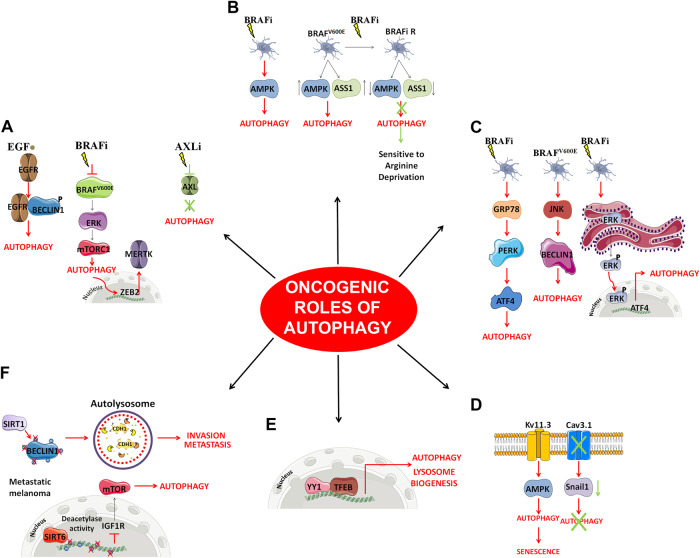
Overview of the main pro-oncogenic mechanisms through which autophagy favors CM progression. Oncogenic mechanisms are depicted in red, whereas suppressor mechanisms are in green. The figure was partly generated using Servier Medical Art, provided by Servier, licensed under a Creative Commons Attribution 3.0 unported license.

In the last decade, several studies have shown that autophagy promotes the growth and survival of CM cells that constitutively expressed the active BRAF^V600E^ mutation, and contributes to resistance to BRAFi/MEKi through several pathways [27, 32, 33], as described below (Table 1) (Fig. 2).

**Table 1:** Pro-tumor role of autophagy in BRAF-mutant CM.

Functional pathway	Gene ID	Function/mechanism	Cell lines	BRAF/NRAS mutational status	Reference
MAPK/ERK pathway	EGFR	EGFR phosphorylated beclin-1 to initiate the autophagic process	M14 WM793	BRAF^V600E^	[38]
	MERKT	The autophagy/MERTK axis desensitized CM cells to BRAFi-triggered apoptosis	A375p A2058 C32 MalMe-3M LAU-T672E LAU-Me246.M1 LAU-T392E LAU-T387B MalMe SK-Mel-19 SK-Mel-29 SK-Mel-100 WM35 WM39 WM278 WM793b WM1552c	BRAF^V600E^	[39]
			SK-Mel-2	NRAS^Q61L^	
			Mel-1300 GR4 DETT-Mel SK-Mel-23 NA8 HBL	BRAF/NRAS wt	
	AXL	AXL targeting inhibited autophagy and improved BRAFi efficacy	A375 Melmet 1 Melmet 5 LOX SK-Mel-28 WM983b	BRAF^V600E^	[40]
			WM239	BRAF^V600D^	
			WM1366	NRAS^Q61L^	
			WM45.1	BRAF/NRAS wt	
AMPK pathway	AMPK	AMPK-dependent autophagy activation was found to be essential for BRAF-mutant CM cells to develop resistance to targeted therapy	YUKSI YUSIK YUGEN8	BRAF^V600E^	[44]
			YUSIT1	BRAF^V600K^	
			YUSIV YUVON	BRAF wt	
			A375 WM35 SK‐Mel-28	BRAF^V600E^	[30]
	AMPK-α1	CM cells initially upregulate AMPK-α1and autophagy to survive, but the establishment of resistance led to AMPK-α1 dowregulation and a metabolism switch from glucose toward arginine dependence	A375 A2058 UACC62 SK‐Mel-28 Mel‐1220 Mel‐DA	BRAF^V600E^	[46, 45]
ER stress	GRP78	BRAFi promoted the interaction between BRAF^V600E^ and GRP78 thus activating ER-stress response and autophagy	A375P SK-Mel-5 1205Lu Mel-624 Mel-1617 WM983B	BRAF^V600E^	[50]
	JNK	BRAF^V600^ fueled a chronic ER stress state that induced autophagy through JNK induction	SK-Mel-5 SK-Mel-28 MeWo G-361 A375 A2058	BRAF^V600E^	[51]
			CHL-1 SK-Mel-110	BRAF wt	
	ERK	Following treatment with BRAFi/MEKi ERK activated the nuclear ER stress response transcription factor ATF4	WM-3936 WM-4231 WM-3629 WM-3670 WM-1963	BRAF/NRAS mut	[53]
			WM-239A	BRAF^V600D^	
			A375P WM-3912	BRAF^V600E^	
			WM-3918 WM-4205 WM-4262 WM-3960	BRAF/NRAS wt	
Plasma membrane channels	Kv11.3	Kv11.3 induced autophagy via activation of an AMPK-dependent signaling pathway	A375	BRAF^V600E^	[56]
	Cav3.1	Cav3.1 inhibition impaired autophagy and decreased the expression of Snail1 along with the motility and invasion ability of BRAF-mutant CM cells	M3 M238 M249 A375M M36 UACC257	BRAF^V600E^	[57]
			TPR WM-1366 Sk-Mel-147	NRAS^Q61H^	
TFEB	YY1	YY1 acted as a cofactor of TFEB to contribute to autophagy regulation. YY1 inhibition reduced autophagy and sensitized BRAF-mutant CM cells to vemurafenib treatment	A375 G-361	BRAF^V600E^	[62]
Sirtuins	SIRT1	SIRT1 deacetylated beclin-1 thereby increasing autophagy and autophagic degradation of E-cadherin	Sk-Mel-28 A375	BRAF^V600E^	[64]
	SIRT6	SIRT6 reduced cell survival, and led to cell-cycle arrest and apoptosis in primary BRAF-mutant CM, but also protected the metastatic BRAF-mutant CM from apoptosis	WM793B WM35 451Lu A2058 Hs 294 T A375	BRAF^V600E^	[66]
			WM266-4	BRAF^V600D^	

*CM* cutaneous melanoma, *ER* endoplasmic reticulum, *EGFR* Epidermal growth factor receptor, *GRP78* glucose-regulated protein, *JNK* c-Jun N-terminal kinase, *PERK* protein kinase RNA-like endoplasmic reticulum kinase, *ATF4* activating transcription factor 4, *PI3K* phosphoinositide 3-kinases, *mTOR* mammalian target of rapamycin, *AMPK* AMP-activated protein kinase, *SNAIL* zinc finger protein SNAI1, *MAPK* mitogen-activated protein kinase, *EGFR* epidermal growth factor receptor, *BRAFi* BRAF inhibitor, *YY1* Yin Yang 1, *SIRT* sirtuin.

#### MAPK signaling pathway (Fig. 2A)

Receptor tyrosine kinases (RTKs) have long been demonstrated to play key roles in BRAF-mutant CM development and resistance to targeted therapy through enhancing MAPK signaling cascade (for review see [34–37]). Intriguingly, Iuliis et al. reported that the RTK epidermal growth factor receptor (EGFR), once phosphorylated, activated beclin-1 to initiate the autophagic process in the BRAF-mutant M14 and WM793 CM cell lines [38]. Besides EGFR, members of the Tyro3-Axl-MERTK RTK family are often aberrantly expressed in BRAF-mutant CM. In this context, MERTK appeared to be stringently regulated by the autophagy signaling pathway, and the autophagy/MERTK axis desensitized CM cells to BRAFi-triggered apoptosis. Notably, dual-inhibition of BRAF^V600E^ and autophagy not only inhibited MERTK expression, but also significantly reduced tumor burden in xenograft mice [39]. While Xue et al. have shown that autophagy contributed to MERTK activation, Nyakas et al. found that AXL signaling promoted a drug-resistant phenotype by inducing autophagy in BRAF-mutant CM. Consistently, AXL targeting improved the BRAFi-induced apoptosis, stimulated ferroptosis and inhibited the autophagic flux [40].

#### AMP-activated protein kinase signaling pathway (Fig. 2B)

AMP-activated protein kinase (AMPK) represents a central regulator of metabolism and autophagy (for review see [41]). AMPK not only phosphorylates ULK1, but also indirectly activates ULK1 by suppressing mTOR signaling [42, 43]. Activation of AMPK-dependent autophagy was found to be essential for the survival of BRAF-mutant CM cells and for the resistance to treatment with both BRAFi [44] and MEKi [30]. Intriguingly, Ying-Ying Li et al. reported that treatment with BRAFi initially triggered autophagy to counteract cell death, but the establishment of resistance to BRAFi was characterized by a substantial attenuation of autophagy-associated proteins, including AMPK-α1, which represents the catalytic subunit of AMPK protein [45]. The same group further demonstrated that AMPK-α1 down-regulation not only abrogated autophagy, but also switched the metabolism from glucose toward arginine dependence [46]. Following arginine deprivation, normal CM cells were found to up-regulate AMPK-α1 and autophagy along with argininosuccinate synthetase (ASS1) to synthesize arginine. Differently, BRAFi-resistant CM showed low or no expression of ASS1 protein, so they required exogenous arginine for survival [45]. Therefore, BRAFi-resistant CM cells were very sensitive to ADI-PEG20, a mycoplasma enzyme degrading arginine to citrulline and ammonia [45]. Overall, these findings provide opposite evidences regarding the role of AMPK in CM; hence, further investigations are required to better define AMPK role in CM progression.

#### Endoplasmic reticulum stress (Fig. 2C)

Prolonged accumulation of misfolded proteins was found to disrupt endoplasmic reticulum (ER) function resulting in ER stress and unfolded protein response (UPR) [47–49]. In a study by Ma et al., cytoprotective autophagy has been shown to be triggered via the ER stress response in BRAF-mutant CM patients and cell lines treated with BRAFi, thus identifying autophagy as a potential adaptive resistance mechanism to targeted therapy. Mechanistically, BRAFi promoted the interaction between BRAF^V600E^ and the molecular chaperone Glucose regulated protein 78 (GRP78), a master regulator of ER stress. More precisely, BRAFi induced the translocation of BRAF^V600E^ into the ER where the mutant BRAF sequestered GRP78 in order to relieve the GRP78-mediated inhibition of PKR-like ER-kinase (PERK), one of the major ER stress sensor. Once activated, PERK phosphorylated the eukaryotic initiation factor 2α and increased the expression of the activating transcription factor 4 (ATF4), which is known to regulate LC3 expression. Notably, co-inhibition of BRAF and PERK completely abrogated autophagy, highlighting the crucial role of PERK in BRAFi-induced autophagy [50]. In line with these concerns, Corazzari et al. confirmed that BRAF^V600E^ induced chronic ER stress in order to up-regulate the basal autophagy flux and to prevent the ER stress-induced apoptosis in CM. More precisely, BRAF^V600E^-mediated ER stress response stimulated c-Jun N-terminal Kinase (JNK), which in turn phosphorylated the inhibitory partners of beclin-1, Bcl-XL and Bcl-2, triggering the release of beclin-1 and autophagy induction. The same authors further demonstrated that treatment of the BRAF^V600^ overexpressing SK-Mel-110 cells with 4-phenylbutyric acid significantly reduced ER stress along with autophagy, and improved the efficacy of a panel of pro-apoptotic drugs [51, 52]. Subsequently, Ojha et al. provided additional insight into the crosstalk between the ER stress and autophagy in promoting resistance to targeted therapies in BRAF-mutant CM. In detail, authors reported that following CM treatment with BRAFi/MEKi, several members of the MAPK pathway translocated into the ER through the cooperative association between GRP78 and the scaffolding protein kinase responsive to stress 2 (KSR2) [53]. Interestingly, ERK was then translocated into the cytoplasm and phosphorylated by PERK. Once reactivated, ERK finally stabilized the nuclear ER stress response transcription factor ATF4, thereby promoting cytoprotective autophagy both in vitro and in vivo [53].

#### Plasma membrane channels (Fig. 2D)

Plasma membrane channels have been widely implicated in autophagy control (for review see [54, 55]). For instance, the pharmacological activation of the plasma membrane voltage-gated potassium channel Kv11.3 with the small molecule NS1643 was found to induce autophagosomes formation via activation of an AMPK-dependent signaling pathway in BRAF-mutant A375 CM cells. In addition to autophagy, stimulation of Kv11.3 channel with NS1643 promoted senescence as well, suggesting that autophagy might represent a survival mechanism for the acquisition of a cellular senescence phenotype. Interestingly, co-treatment of A375 cells with hydroxychloroquine (HCQ), an autophagy inhibitor, and NS1643 led to apoptosis as indicated by caspase-3 cleavage. Despite these findings, however, the exact molecular mechanism of Kv11.3 in controlling AMPK is yet to be elucidated [56].

Among the T-type calcium channels (TTCCs), high levels of Cav3.1 and Cav3.3 mRNAs were measured in BRAF-mutant CM [57]. Interestingly, the expression of the TTCC Cav3.1 isoform was significantly correlated to the autophagic status in BRAFi-resistant CM tissues and cells, and in silico analysis revealed an enrichment of Cav3.1 expression in CM following treatment with BRAFi [57]. Notably, TTCC targeting by small interfering RNA or by specific inhibitors impaired the autophagic flux, thus reducing proliferation and promoting apoptosis in BRAF-mutant CM cells [58]. In addition, TTCC inhibition decreased the expression of Snail1 along with the motility and invasion ability of BRAF-mutant CM cells, indicating that therapeutic strategies targeting TTCCs and autophagy might inhibit CM progression and metastasis probably by decreasing Snail1 expression [57]. More importantly, preclinical studies suggested that TTCC blockers might represent an alternative therapeutic strategy to reduce tumor growth by autophagy blockade in resistant BRAF^V600E^-mutant CM as well [59].

#### Transcription factors (Fig. 2E)

The zinc-finger transcription factor, Yin Yang 1 (YY1) has been shown to be a critical regulator of many basic biological processes and to act as an activator or a repressor depending on its spatial and temporal context (for review see [60]). Interestingly, Du et al. identified YY1 as a cofactor of the transcription factor EB (TFEB), which is known to regulate the transcription of genes involved in autophagy [61, 62]. The same group further demonstrated that YY1 suppression in BRAF-mutant CM contributed to improved antitumor efficiency of the BRAFi vemurafenib both in vitro and in vivo, by limiting autophagy and lysosomal functions [62].

#### Sirtuins (Fig. 2F)

Sirtuins are histone deacetylase enzymes that dynamically regulate several cellular processes, including autophagy (for review see [63]). For instance, sirtuin 1 (SIRT1) has been described to deacetylate beclin-1 thereby increasing autophagy and autophagic degradation of E-cadherin, thus promoting the metastatic potential of BRAF-mutant CM cells [64]. Besides SIRT1, SIRT6 has also been found to modulate the autophagic process in BRAF-mutant CM [65, 66]. In this context, a recent study by Wang et al. highlighted the complex role of autophagy in CM. In fact, basal levels of autophagy were low in primary CM when compared to benign nevi, but aberrantly up-regulated in CM metastatis. Additionally, Wang and colleagues reported that SIRT6 overexpression reduced cell survival, and led to cell-cycle arrest and apoptosis in the primary BRAF-mutant CM cell lines WM35 and WM793B, but also protected the metastatic BRAF-mutant CM cell lines A2058 and A375 from apoptosis [66]. Of note, the treatment with the autophagy inhibitor chloroquine (CQ) partially rescued SIRT6 overexpression in primary WM35 and WM793B cells since it promoted cell proliferation and cell cycle progression, and impaired apoptosis, whereas the autophagy activator rapamycin partially reversed the effect of SIRT6 knockdown on metastatic A2058 and A375 cells [66]. These data led authors to conclude that SIRT6 regulated BRAF-mutant CM growth in an autophagy-dependent way. Further mechanistic investigations revealed that the aberrant effects of SIRT6 on autophagy were mediated through the IGF/AKT/mTOR signaling pathway [66]. In fact, deacetylation of histones by SIRT6 prevented the expression of the IGF receptor, thereby reducing AKT and mTOR activities, and promoting autophagy [66].

### Tumor suppressive role of autophagy in BRAF-mutant CM

As abovementioned, autophagy appears to be pro-tumorigenic and essential for BRAF-mutant CM development and progression. Nevertheless, the autophagic factors ATG5 and ATG7 were found to display reduced expression in CM tissues compared to benign nevi [67, 68], thus indicating a tumor suppressive role of autophagy in early-stage CM. By using the BRAF-mutant SK-Mel-5 cell line, Frangež et al. revealed that ATG5 and ATG7 expression was promoted by the transcription factor nuclear respiratory factor 1 (NRF1). However, following NRF1 silencing, the subsequent decrease of ATG5 and ATG7 expression did not impact on autophagy in vitro, thus suggesting that NRF1 might not be the sole driver of autophagy in vivo [67].

Interestingly, the depletion of ATG5 promoted CM initiation by impairing the oncogene-induced senescence in human melanocytes expressing BRAF^V600E^ [69]. The functional relationship between autophagy and senescence was subsequently confirmed by Li et al., since they demonstrated that the BRAFi encorafenib was able to induce senescence along with autophagy activation. More importantly, the co-treatment with encorafenib and autophagy inducers significantly improved the growth inhibition of the BRAF-mutant A375 cells respect to encorafenib alone [70]. The potential tumor suppressive role of ATG5 was further empathized in a study by García-Fernández et al. which showed that heterozygous loss of ATG5 in melanocyte-specific mouse models enhanced CM metastasis and compromised the response to the BRAFi dabrafenib [71].

Of interest, although the molecular mechanisms by which autophagy responds to BRAF signaling has not been completely elucidated yet, Li et al. have correlated the oncogenic BRAF^V600E^ mutation with autophagy inhibition and CM progression. In fact, they reported that BRAF^V600E^ promoted the ERK-mediated phosphorylation and subsequent inactivation of TFEB, which represents the master regulator of the expression of autophagy/lysosomal genes. The inhibition of the transcriptional program triggered by TFEB resulted in elevated CM cell proliferation, TGF-β-mediated epithelial mesenchymal transition (EMT), metastasis formation, and resistance to targeted therapy. On the contrary, TFEB expression activated autophagy and reduced in vivo tumor growth [29].

BRAF^V600E^ was also found to drive liver kinase B1 (LKB1) inhibition through ERK and ribosomal S6 Kinases (RSKs), thus compromising the ability of LKB1 to bind and to activate AMPK [72], which is crucial for autophagy initiation, as described above. Despite these findings, however, the association between AMPK and the autophagy pathway has not been addressed in this study. RSKs are indicated as principal effectors of the MAPK/ERK signaling pathway. Consistently, reactivation of the MAPK/ERK cascade following BRAFi acquired resistance reinforced RSK activity in CM cells. Intriguingly, Xu Zhang et al. demonstrated that RSK2 knockout or pharmacological inhibition reduced cell proliferation and metastasis, and significantly augmented the ratio of LC3-II/LC3-I, an indicator of increased autophagy flux, thus suggesting that RSK2 might promote BRAF-mutant CM progression by inhibiting autophagy [73].

## Role of mitophagy in BRAF-mutant CM

Mitochondria are double-membrane subcellular organelles essential for the control of energy homeostasis given their role in the production of ATP through oxidative phosphorylation (OXPHOS) [74]. Apart from energy production, mitochondria play crucial roles in a range of fundamental processes, including reactive oxygen species (ROS) production, heme and steroid hormones biosynthesis, iron homeostasis, and apoptosis. Since dysfunctional or damage mitochondria can be detrimental to cellular homeostasis, several quality control mechanisms have evolved to restore and to preserve energy metabolism, including mitophagy. The term mitophagy refers to the controlled degradation of mitochondria via the autophagosome-lysosome pathway [14, 74]. Mitophagy impairment triggers the accumulation of defective mitochondria, whereas excessive mitophagy may lead to cell death due to the loss of functional mitochondria and severe reduction in cellular energy levels. Although this complex process is not yet fully understood, a large number of mitophagy-related proteins have been identified (see review [74, 75]). At present, only few studies have investigated how mitophagy signaling pathway might affect BRAF-mutant CM. For instance, by using two early‐stage CM cell lines, the BRAF wild type 530 and BRAF^V600E^ mutant WM35, Vara‐Pérez et al. hypothesized that the mitophagy-associated receptor BCL-2 interacting protein 3 (BNIP3) facilitated CM development by supporting the stability of the hypoxia inducing factor-1α (HIF‐1α) protein. Intriguingly, BNIP3 itself is a well-known transcriptional target of HIF‐1α, and its expression was found to be rapidly induced under hypoxic conditions in order to promote mitophagy, thus facilitating the tumor metabolic switch from OXPHOS to glicolysis [76]. Nevertheless, whether BRAF^V600E^ mutation specifically affects BNIP3-driven mitophagy is still unknown.

As stated above, autophagy might be induced by ER stress. In vitro studies in the BRAF-mutant A2058 and 451Lu cells demonstrated that ER stress could modulate mitochondrial fission and mitophagy as well. Mechanistically, under ER stress, the unfolded protein response promoted the transcription of the Mitochondrial E3 ubiquitin-protein ligase (MARCH5) to facilitate the ubiquitination and degradation of mitofusin 2 (MFN2), which usually mediates mitochondria fusion, thereby inducing mitochondrial fission and mitophagy. Therefore, CM cells resistant to ER-stress inducing agents displayed high levels of mitochondrial fission and mitophagy which contributed to preserve mitochondrial functions [77].

It has become increasingly clear that targeted therapy deeply alters the metabolism of BRAF-mutant CM cells. More precisely, treatment with BRAFi/MEKi significantly reduces glucose uptake and suppresses glycolysis in CM cells, thus stimulating mitochondrial activity and OXPHOS dependency for survival [78, 79]. Therefore, targeting mitochondrial functions likely represents an appealing therapeutic strategy to overcome resistance to BRAFi/MEKi. Consistent with this hypothesis, the inhibition of the OXPHOS mitochondrial complex led to cell death by inducing mitochondrial permeability transition pore opening followed by mitophagy which, in turn, promoted ROS generation and subsequent oxidative stress in BRAF-mutant CM [80–82]. A recent study by Rudolf et al. provided the first evidence that acutely increased intracellular zinc specifically enhanced the mitophagic flux to recover damaged mitochondria in metastatic CM cells. Accordingly, a reduction in mitochondrial membrane potential and ATP production, along with increased superoxide levels, were also observed [83]. Therefore, although this study was conducted in CM cultures with unknown BRAF status, we might speculate that treatment with zinc pyrithione might improve the efficacy of targeted therapy.

## Secretory autophagy in BRAF-mutant CM

Besides its degradative function, autophagy facilitates the secretion of a growing list of tumor proteins. This protein secretion process, which usually bypasses the conventional ER-Golgi route, is called secretory autophagy (SA) (for review see [84]), and adds more complexity to the pleiotropic role of autophagy in controlling homeostasis in both normal and tumor cells. Kraya et al. postulated that the quantity of secreted proteins might reflect the intracellular autophagy level in CM cells harboring BRAF mutation. In fact, by comparing the secretome extracted from low- and high-autophagy BRAF-mutant CM cells, authors observed that a low autophagic activity was associated to a significant decrease in the expression level of interleukin (IL)-1β, C-X-C Motif Chemokine Ligand 8, Leukemia Inhibitory Factor, Family With Sequence Similarity 3 Member C, and Dickkopf WNT Signaling Pathway Inhibitor 3 [85].

Importantly, the unconventional protein secretion through SA was shown to affect therapy responses and to contribute to drug resistance in several tumor types, including CM. In this context, Martin et al. observed that BRAF-mutant resistant CM exhibited increased autophagy-driven ATP secretion that, in turn, activated purinergic receptors along with MAPK and PI3K signaling pathways to promote cell invasion and migration [86]. More recently, Barceló et al. reported that conditioned media secreted from BRAFi-resistant CM cells contributed to the acquisition of a resistance phenotype in sensitive CM cell lines. Further analyses revealed that the secretome of BRAFi-resistant CM cells was rich in angiogenic factors and pro-tumoral cytokines. Among them, the macrophage colony-stimulating factor (M-CSF) played an important role in the development of BRAFi resistance. Consistently, co-treatment with M-CSF monoclonal antibody, vemurafenib, and autophagy blockers synergistically induced apoptosis, impaired migration and reduced tumor growth in BRAFi-resistant CM both in vitro and in vivo [87].

As described above, only few studies have focused on the role of SA in BRAF-mutant CM so far. However, we can postulate that besides to influence tumor cells, molecules released by SA might reprogram CM microenvironment and/or reach the blood vessels to induce systemic modifications that would contribute to BRAF-mutant CM growth and treatment failure.

## Non-coding RNAs involved in autophagy regulation in BRAF-mutant CM

The ENCyclopedia of DNA Elements project revealed that the majority of the human genome is transcribed into RNA. Indeed, it is now widely recognized that only about 1.5–2% of the human genome consists of protein-coding sequences, whereas the rest is composed of non-coding RNAs (ncRNAs) [88–92].

Among the so-called short ncRNAs (<30 nucleotides), microRNAs (miRNAs) represent the most abundant and studied class. In the last decade, miRNAs have been extensively studied and characterized in CM (for review see [93, 94]), and have been demonstrated to modulate autophagy activity in CM harboring the BRAF-mutation as well. In particular, miR-23a was reported to strongly reduce invasion capability of A2058 and A375 BRAF-mutant CM cell lines through ATG12 targeting and relative autophagy inhibition [95]. Accordingly, miR-23a expression was significantly decreased in metastatic CM tissues and in serum of CM patients with metastatic disease [95]. miR-138-5p was also significantly decreased in CM patients compared to healthy control subjects. However, miR-138-5p up-regulation generated opposite effect on autophagy respect to miR-23a. In fact, when expressed, miR-138 inhibited A2058 cell proliferation and induced apoptosis by suppressing the PI3K/AKT/mTOR autophagy signaling pathway [96]. Of interest, miR-138-5p was found to negatively regulate HIF-1α as well, thus impairing BRAF-mutant CM cells migration and invasion in vitro, and their metastatic potential in vivo [97, 98]. In this context, a number of studies have unveiled the molecular connections between autophagy and hypoxia in CM [99–102]. Hence, it would be of interest to address in more detail the specific role of miR-138-5p in regulating the hypoxia/autophagy axis. In addition to miR-23a and miR-138-5p, miR-24-1 might also act as a tumor suppressor since its expression was significantly lower in tissues from primary and metastatic CM lesions, as well as in CM specimens associated with lymph node metastasis [103]. Of note, following miR-24-1 induction, enhanced levels of beclin-1 and LC3-II/I ratio, along with decreased expression of Bcl-2 and Bcl-xL, were detected in BRAF-mutant A375 cells, thus suggesting autophagy and apoptosis activation. Further mechanistic investigations revealed that these effects arose from the miR-24-1-mediated silencing of ubiquitin D [103].

Interestingly, autophagy-regulating miRNAs could also induce sensitivity to targeted therapy in BRAF-mutant cells. For instance, Luo et al. found that miR-216b directly repressed autophagic activity in A375 cells by reducing mRNA and protein expression levels of ATG5, beclin-1, and UVRAG, which is usually associated with the beclin-1/PI3KC3 complex. More importantly, ectopic expression of miR-216b increased the efficacy of vemurafenib both in vitro and in vivo, and sensitized resistant CM cells to BRAFi by inhibiting cytoprotective autophagy [104]. In a similar study, treatment of CM cells with miR-26a mimic improved BRAFi efficacy via targeting the autophagy inducer high mobility group box 1 (HMGB1) [105]. On the other hand, miR-153-3p overexpression sensitized the BRAF-mutant A375 and M14 cells to the treatment with the chemotherapeutic agent decarbazide by negatively regulating ATG5 expression [106]. At the moment, however, it remains to be clarified whether the miR-153-3p/ATG5 axis might affect the response to target therapy as well.

Among oncogenic miRNAs, miR-1246 up-regulation has been related to BRAFi resistance in multi-drug resistant A375P CM cells. Although the exact mechanism has not been completely identified, authors speculated that resistance to BRAFi in miR-1246 mimic-transfected cells was mostly due to autophagy inhibition in order to allow CM cells to escape cell death following vemurafenib treatment [107].

**Fig. 3:**
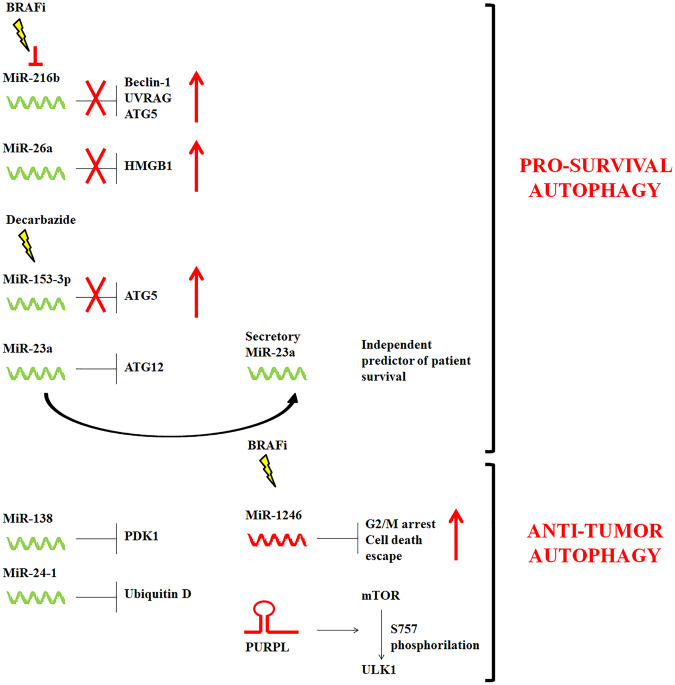
Non-coding RNAs involved in autophagy regulation in BRAF-mutant CM. Suppressor non-coding RNAs are depicted in green, whereas oncogenic non-coding RNAs are in red.

Besides miRNAs, long non-coding RNA (lncRNAs) (>200 nucleotides) have emerged as critical regulators of several intracellular processes in BRAF-mutant CM [88, 91, 108]. In addition, there is increasing evidence that lncRNAs might modulate the expression of different autophagy associated proteins in cancer cells (Fig. 3) [109, 110]. Despite these findings, however, only the autophagy-related lncRNA p53 upregulated regulator of p53 levels (PURPL) was found up-regulated in CM tumors and in the BRAF-mutant CM cell lines A375, SK-Mel-1, and SK-Mel-28 [111]. Consistently, PURPL depletion significantly decreased proliferation, migration and invasiveness of A375, SK-Mel-1, and SK-Mel-28 cells. Intriguingly, Han et al. found that PURPL physically interacted with ULK-1 and mTOR to promote mTOR-dependent phosphorylation of ULK-1 at Ser 757, thus inhibiting autophagic cell death [111].

## Treatment perspectives

In the last years, the combination of autophagy inhibitors and activators has emerged as a promising new therapeutic option for rewiring aggressiveness of BRAF-mutant CM. Autophagy activators usually stimulate the cellular energy sensor AMPK. Consistently, Lai et al. indicated that the treatment of the BRAF-mutant A375 cells with Panduratin-A (PA) induced autophagy via AMPK activation and mTOR inhibition. More interestingly, pre-treatment with the autophagy inhibitor 3-methyladenine improved the apoptotic-inducing effect of PA on A375 cells, as evidenced by the increased level of PARP cleavage [112]. Subsequently, Ranieri et al. observed that N6-isopentenyladenosine coordinately induced autophagosome accumulation through AMPK activation and inhibition of the prenylation of Rab7, a small GTPase required for autophagosome maturation and fusion with lysosomes. The accumulation of autophagic vacuoles blocked the autophagic flux, thus inducing apoptosis in BRAF-mutant A375 cells in vitro and in xenografts models, and affected the response to targeted therapy [113].

PI3K/AKT/mTOR signaling pathway has been found to enable BRAF-mutant CM development and to promote resistance to targeted therapy [114–116]. Interestingly, PI3K/AKT/mTOR targeting has been assessed in combination with autophagy inhibition. In fact, Xie et al. observed that treatment with the mTOR inhibitor temsirolimus (TEM) induced autophagy in a panel of BRAF- and NRAS-mutant CM cell lines, thus limiting its efficacy. Interestingly, co-treatment with HCQ impaired the autophagic flux, and autophagosomes accumulation led to reduced tumor growth and apoptosis both in vitro and in vivo [115]. These results suggested that CM might be treated through coordinate autophagy and mTOR inhibition. On this ground, the phase I clinical trial NCT00909831 aimed to evaluate the maximum tolerated dose, safety, preliminary activity, pharmacokinetics, and pharmacodynamics of HCQ in combination with TEM. In this phase I study, that was carried out in patients with stage IV CM, stable disease was observed in 74% of patients treated with TEM and HCQ combination. However, most of the patients enrolled in this study were BRAF wild-type [114]. The subsequent phase I clinical trial NCT01480154 analyzed the efficacy of combining HCQ with dose-intense temolozide, an alkylating chemotherapeutic drug [117]. This study showed encouraging results, since one elderly metastatic CM patient had a near-complete response, whereas a patient with metastatic CM, who had failed 2 prior therapies, had stable disease for over 8 months [117]. However, mutational status of the tumor was missing for most of the patients, and there was no correlation between TMZ and HCQ activities and BRAF mutation.

In addition to impair tumor growth, autophagy inhibitors have been shown to improve targeted therapy efficacy and to overcome drug resistance. These data led to the BRAF Autophagy and MEK Inhibition in Melanoma (BAMM) phase I/II clinical trial in which HCQ has been tested in a group of 34 unresectable stage III or stage IV BRAF-mutant CM patients in combination with the BRAFi dabrafenib and the MEKi trametinib (BAMM: NCT02257424). BAMM trial enrolled a high percentage of BRAF-mutant CM patients with prior immunotherapy, large tumor size at baseline, and elevated levels of serum lactate dehydrogenase (LDH), which is a well-known marker of poor outcome in CM. Results from this trial were recently published and, interestingly, in patients with elevated LDH the response rate was 88%, whereas the median progression-free survival (PFS) and overall survival were 7.3 and 22 months, respectively, thus suggesting this regimen might be effective for eliciting responses in the most aggressive BRAF-mutant CM cases [118]. Notably, RNA-seq analysis indicated that, among autophagy genes, ATG12 and BNIP3 were significantly up-regulated in the pre-treatment tumors of patients with short PFS [118]. Another phase I/II clinical trial (NCT03754179) was recently conducted with the aim to investigate the use of combined BRAFi/MEKi plus HCQ in patients with unresectable stage III or stage IV BRAF^V600^-mutant CM who previously progressed on prior treatment with BRAFi/MEKi and immune checkpoint inhibitors [119]. Unfortunately, the recruitment was prematurely closed due to a negative evaluation of the risk/benefit ratio for adding HCQ associated to BRAFi/MEKi rechallenge [119]. Other trials are ongoing to better define the advantage to inhibit autophagy for the treatment of BRAF-mutant CM.

## Conclusions and perspectives

In the last years, extensive studies have indicated that although autophagy might have contradictory or context-dependent roles, its dysregulation is closely related to tumorigenesis and progression in CM harboring the BRAF mutation. Hence, since most of these studies have been conducted by using in vitro or in vivo models, it would be important to identify the autophagy-related markers with predictive or prognostic potential in BRAF-mutant CM. In addition, future investigation is warranted to assess the therapeutic potential of autophagy modulators in order to overcome resistance to current targeted approaches. To this end, considering that advanced CM patients are often treated with first-line immune checkpoint inhibitors, another major issue to further clarify is how autophagy modulates anti-tumor immune response in BRAF-mutant CM.

## Data Availability

Data sharing not applicable to this article as no datasets were generated or analysed during the current study.
